# Critical influence of the thymus on peripheral T cell homeostasis

**DOI:** 10.1002/iid3.132

**Published:** 2016-11-28

**Authors:** Pedro Henrique Oliveira Vianna, Fábio B. Canto, Jeane S. Nogueira, Caroline Fraga Cabral Gomes Nunes, Adriana César Bonomo, Rita Fucs

**Affiliations:** ^1^ Departamento de ImunologiaInstituto de Microbiologia Paulo de Goés (IMPG)—Universidade Federal do Rio de JaneiroRio de Janeiro—RJBrazil; ^2^ Departamento de ImunobiologiaInstituto de Biologia—Universidade Federal FluminenseNiterói—RJBrazil; ^3^ Programa FIOCANCERVPPLR—Instituto Oswaldo Cruz—FIOCRUZRio de Janeiro—RJBrazil

**Keywords:** Peripheral T cell homeostasis, regulatory T cells, thymic output

## Abstract

**Introduction:**

A tight balance between regulatory CD4^+^Foxp3^+^ (Treg) and conventional CD4^+^Foxp3^−^ (Tconv) T cell subsets in the peripheral compartment, maintained stable throughout most of lifetime, is essential for preserving self‐tolerance along with efficient immune responses. An excess of Treg cells, described for aged individuals, may critically contribute to their reported immunodeficiency. In this work, we investigated if quantitative changes in thymus emigration may alter the Treg/Tconv homeostasis regardless of the aging status of the peripheral compartment.

**Methods:**

We used two different protocols to modify the rate of thymus emigration: thymectomy of adult young (4–6 weeks old) mice and grafting of young thymus onto aged (18 months old) hosts. Additionally, lymphoid cells from young and aged B6 mice were intravenously transferred to B6.*RAG2^−/−^* mice. Alterations in Treg and Tconv peripheral frequencies following these protocols were investigated after 30 days by flow cytometry.

**Results:**

Thymectomized young mice presented a progressive increase in the Treg cell frequency, while the grafting of a functional thymus in aged mice restored the young‐like physiological Treg/Tconv proportion. Strikingly, T cells derived from young or aged splenocytes colonized the lymphopenic periphery of RAG^−/−^ hosts to the same extent, giving rise to similarly elevated Treg cell levels irrespective of the age of the donor population. In the absence of thymus output, the Treg subset seems to survive longer, as confirmed by their lower proportion of Annexin‐V^+^ cells.

**Conclusions:**

Our data suggest that the thymus‐emigrating population, harboring an adequate proportion of Treg/Tconv lymphocytes, may be essential to keep the Treg cell balance, independently of age‐related shifts intrinsic to the peripheral environment or to the T cell biology.

## Introduction

Peripheral frequencies of T lymphocytes are very tightly regulated. Total numbers of T cells are maintained stable over lifetime, despite permanent thymic emigration or following perturbations of the homeostatic status, such as transitory states of lymphopenia or lymphoproliferation. Thymus output contributes with a constant renewal of the TCR repertoire, delimited by the competition for survival between recently thymus‐emigrated (RTE) and mature resident T cells. An adequate balance of regulatory and effector T cells with different functional profiles are also physiologically preserved under steady‐state in the secondary lymphoid organs. Interactions with the peripheral universe of peptides, including not only self‐peptides but also those derived from the microbiota, are essential to the long‐term persistence of each T cell clone, to maintain a diverse polyclonal TCR repertoire and may also interfere in the functional plasticity of the T lymphocyte population 1, 2, 3.

CD4^+^Foxp3^+^ regulatory T (Treg) cells, kept as 5–10% of the peripheral T lymphocytes during most adult life, comprises two different major subsets: the natural thymic‐derived Treg cells (tTreg), selected through high‐affinity interactions with self‐peptides presented by the thymic stroma and usually characterized by Helios and neuropilin‐1 (Nrp‐1) expression; and the peripherally induced regulatory T cells (pTreg), derived from naïve Foxp3‐negative lymphocytes after TCR stimulation in the presence of adequate cytokine stimulation 4, 5, 6, 7, 8, 9. Each of these subsets encompasses a unique and complementary TCR repertoire, both necessary to control several autoimmune pathologies 10, 11, 12.

Treg cells also display a previously unappreciated wide functional plasticity, influenced by the cytokine milieu, in response to the local microenvironment. The “naïve” and “effector/memory” phenotypes of Treg cells have been recently characterized 13, 14, 15, as well as different subsets of “inflammatory” or “polarized” Treg cells, which co‐express other lineage‐specific transcription factors besides expressing Foxp3 16, 17, 18. As well characterized for human Treg cells, the presence of T‐bet and RORγt results in the expression of CXCR3 and CCR6, respectively, which help address them to particular sites of inflammation where special types of effector T cells accumulate 19. Different intensities of CD25 expression among Foxp3^+^ lymphocytes were also described, leading to the characterization of CD25^neg/low^ and CD25^high^ subtypes of Treg cells 20. The efficiency for peripheral conversion of naïve T cells to the Treg phenotype, and the relative frequency of those several Treg functional subtypes described, may be modified at different ages 21, 22, 23, 24. The contribution of changes in the thymus output versus alterations in peripheral conversion and/or expansion of resident cells, to the Treg cell homeostasis in different periods of life, however, has not been conclusively established yet.

Aged individuals present a significant enhancement in the total percentage of peripheral Treg cells, especially of the CD25^neg/low^Foxp3^+^ 21, 25 and the CD44^+^CD62L^low^Foxp3^+^ subsets 26. A higher intra‐thymic generation of Treg cells in the aged atrophied thymus was already reported 27, although their export to the peripheral RTE cell pool was not confirmed in order to justify the increased Treg cell frequency. A low efficiency for Treg conversion in aged mice was observed 23 and the accumulated Foxp3^+^ cells in the aged periphery are apparently enriched for Helios^+^Nrp1^+^ lymphocytes, supporting their predominant intra‐thymic origin 26. Additionally, an enhanced survival capacity was reported for aged Treg cells, in comparison to those present in young animals, being attributed to a lower expression of the pro‐apoptotic molecule Bim influenced by higher levels of ICOS, CD69, and IL‐6 22, 25, 26. It was not clear, however, if the age‐dependent accumulation of these Bim^low^CD25^low^Foxp3^+^ cells requires the influence of an aged periphery or may result from the interruption of thymus output.

Stable increments in the peripheral Treg cell frequencies may not be a property exclusive of the aged individual. We have recently characterized a higher proliferative potential of Treg cells in comparison to conventional T lymphocytes (Tconv), which enables the former to better colonize lymphopenic peripheral compartments even at a young age 28. In young lymphopenic hosts, such as athymic or RAG^−/−^ mice, the increased proliferative capacity and decreased apoptosis level within the adoptively transferred Treg cell subset lead to an enriched frequency of Foxp3^+^ cells 28, 29 that is very similar to that observed in aged mice. These results might derive from disturbing effects provided by the lymphopenic conditions of the host and/or from the dysfunctional thymus output, which prevents the constant dilution of the peripheral population with a physiologically balanced proportion of newly generated Treg and Tconv lymphocytes.

In the present paper, we used two different protocols to modify the rate of thymus emigration and study the influence of thymic output on the peripheral Treg cell homeostasis: thymectomy of adult young mice and grafting of young thymus onto aged hosts. Our results show that peripheral T cell homeostasis is promptly disturbed in the absence of the thymus. This disturbance was characterized by a preferential persistence of Treg cells that occurs independently of the age of either the T cells or the peripheral environment. The excess of Treg cells in aged mice is also very rapidly corrected by the grafting of a young functional thymus, supporting the hypothesis that thymus newly emigrated T cell populations, harboring an adequate physiological proportion of Treg/Tconv lymphocytes, are essential to compensate for an excess of peripheral Treg cell expansion or survival. The vastly described immunosenescence associated with aging, in which an excess of Treg cells may impair the immune response to infections and tumors, highlights the relevance of understanding the peripheral Treg cell homeostasis for the development of adequate clinical strategies.

## Materials and Methods

**Mice**

Four‐to‐six weeks old (young) or 18 months old (aged) C57BL/6 (B6), C57BL/6.Ba.Thy‐1.1^+^ (B6.Ba), and BALB/c mice were subjected to thymectomy or thymus grafting or used as donors of lymphoid cells in transfer protocols. Five‐to‐ten days old BALB/c or B6.Ba mice were used as donors of thymus. B6 *RAG2^−/−^* mice were used as syngeneic hosts of lymphoid cell transfers. All animals were bred under specific pathogen‐free conditions at NAL/UFF, Niterói, Brazil. All the experimental protocols were approved by the UFF Ethics Committee for Animal Experimentation.

### Adoptive cell transfers

Sterile spleen single‐cell suspensions, obtained through mechanical disruption, from young and aged B6 mice were diluted in phosphate‐buffered saline (PBS). Cells were counted in the presence of Trypan blue and injected intravenously into B6.*RAG2^−/−^* mice (15–20 × 10^6^ cells per animal). The recipient mice were euthanized either 30 or 60 days post‐transfer, and single‐cell suspensions from blood, spleen, and peripheral lymph nodes were stained for FACS analysis.

### Thymus transplantation

Surgery was performed under sterile conditions after intraperitoneal administration of the anesthetics ketamine (100 mg/kg) and xylazine (10 mg/kg) (Dopalen, Ceva, SP) to aged BALB/c or B6 host mice. A dorsolateral incision allowed the exposure of the kidney, where a small hole was made in the organ capsule. Thymic lobes from 5 to 10 days old syngeneic donors were placed under the kidney capsule and the incision was closed with sterile sutures. The grafted thymus was analyzed for different subsets of T cells 30 days after the transplantation.

### Thymectomy

At 4–6 weeks of age, mice were anesthetized and the thymus was removed by suction through a small upper sternal incision. Efficiency of thymectomy was confirmed by visual inspection at the time of euthanasia.

### FACS analysis

Immunofluorescence staining of blood, spleen, peripheral lymph nodes (pooled inguinal and axillary—PLN) and thymus single‐cell suspensions was performed using the following monoclonal antibodies purchased from eBioscience (San Diego, CA) or Biolegend (San Diego, CA): APC, PE/Cy7 or FITC‐anti‐CD4 (GK1.5), PE‐anti‐CD8b (H35‐17.2), PE‐anti‐CD25 (PC61.5), APC or AlexaFluor488‐anti‐Foxp3 (FJK‐16), PE‐anti‐CD44 (IM7), APC‐anti‐CD62L (MEL‐14), and PE‐anti‐neuropilin‐1(3E12). Fluorochrome‐conjugated PECy7‐streptavidin was used along with biotinylated monoclonal antibody anti‐Foxp3. Foxp3 intracellular staining was performed according to eBioscience commercial kit instructions. Stained cells were analyzed on AccuriC6 flow cytometer (BD Biosciences, Franklin Lakes, NJ) with FlowJo software (Treestar, Ashland, OR) version 8.7.

### Apoptosis assay

Spleen cells were incubated with Annexin‐V (Alexa Fluor 647; Biolegend) diluted in specific binding buffer (Invitrogen) for 15 min and resuspended in the same specific binding buffer for analysis in the flow cytometer.

### Statistical analysis

Data were analyzed with software GraphPad Prism 5 (San Diego, CA) by Student's *t*‐test or ANOVA (indicated in figure legends) and considered statistically significant when *p *< 0.05. All graphs show mean and standard deviations.

## Results

Aged mice from our animal facility showed the characteristic alterations already described for CD4^+^ and CD4^+^Foxp3^+^ T cell frequencies in the peripheral compartments 21, 22, 25, 26, 30, 31, 32. As shown in Figure S1, significant lower total CD4^+^ and higher CD4^+^Foxp3^+^ cell frequencies were observed in blood and different secondary lymphoid tissues of 18‐month‐old mice of both strains studied (BALB/ and C57BL/6), in comparison with 4–6‐week‐old counterparts. An increase in effector/memory‐like CD4^+^ cells (CD44^high^CD62L^low^) was also confirmed in aged animals; CD44^high^CD62L^low^ was also the predominant phenotype of Treg cells (“effector” Treg cells). No alterations were seen in the CD8^+^ T cell population.

In order to study the influence of thymic involution on these age‐related shifts, we used two different protocols to modify the rate of thymus emigration: thymectomy of young mice and grafting of young thymus in aged hosts. Alterations in the frequencies of CD4^+^, CD8^+^, CD25^+^Foxp3^+^, and CD4^+^CD44^+^ cells were followed in both groups of animals.

### Interruption of thymus output leads to increased Treg/Tconv ratio in the peripheral compartment

**Figure 1:**
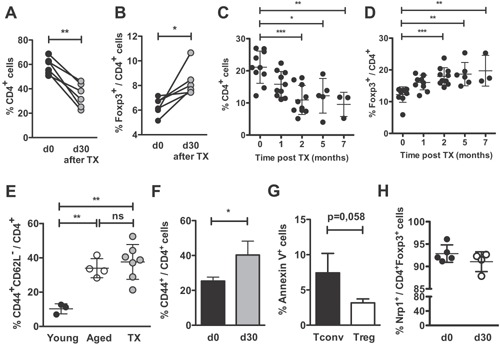
Alterations in peripheral T cell populations after thymectomy simulates those observed in aged mice. BALB/c or C57BL/6 mice were thymectomized at the age of 4–6 weeks (Tx). Frequencies of (A) CD4^+^ and (B) Foxp3^+^/CD4^+^ in BALB/c blood samples are shown right before and 30 days after thymectomy. Open and filled circles connected by a line represent one individual mouse. (C–D) Peripheral blood frequencies of (C) CD4^+^ and (D) Foxp3^+^/CD4^+^ T cells were followed over 7 months after Tx in B6 mice (*n* = 10 at months 1 and 2, *n* = 5 at month 5, and *n* = 3 at month 7). (E) Percentages of CD44^+^CD62L^low^/CD4^+^ cells in the blood of young (2 months old; *n* = 9; black filled circles), aged (>18 month old; *n* = 4; open circles) and Tx (*n* = 6; gray filled circles) BALB/c mice. (F) Frequencies of CD44^+^/CD4^+^ are shown before and 30 days after Tx in B6 mice (*n* = 7). (G) Percentage of annexin‐V^+^ cells among CD4^+^Foxp3^−^ and CD4^+^Foxp3^+^ subsets after thymectomy (*n* = 5). (H) Frequencies of neuropilin‐1^+^/CD4^+^Foxp3^+^ are shown before and 30 days after Tx in B6 mice (*n* = 5). Each circle represents one individual mouse. Data are pooled from three independent experiments and statistical significance was determined by paired Student's *t*‐test (A and B), one‐way ANOVA with Bonferroni's Multiple Comparison Test (C–G) and unpaired Student's *t*‐test (H) (*p < 0.05, **p < 0.01, ***p < 0.001; ns, not significant).

At the age of 2–4 weeks, mice of both strains were thymectomized and their CD4^+^, CD4^+^Foxp3^+^, and CD4^+^CD44^+^ cell frequencies were analyzed in blood samples up to 7 months after surgery. The interruption of thymus output in the thymectomized young animals resulted in lower numbers of CD4^+^ (Fig. 1A) and higher CD4^+^Foxp3^+^ (Fig. 1B) T cell frequencies in all mice studied, simulating the effects of natural thymus involution in aged mice. These alterations, already detectable as soon as 30 days after thymectomy, were maintained for at least 7 months (Fig. 1C,D). The CD44^+^CD62L^−^ phenotype was also very similar to that verified in aged mice and significantly different from that of non‐manipulated young animals (Fig. 1E,F).

Absolute numbers of total CD4^+^ and CD4^+^Foxp3^−^ cells were decreased in the blood of thymectomized mice, while those of CD4^+^Foxp3^+^ cells were not significantly modified (Fig. S2), suggesting that the relative increase in Treg cell frequencies was a consequence of their longer survival in the periphery compared to Tconv lymphocytes. Supporting this interpretation, the percentage of annexin‐V^+^ cells was indeed lower within the CD4^+^Foxp3^+^ subset than within the Tconv population, before (not shown) and after thymectomy (Fig. 1G).

We wondered whether the accrual of Treg cells in thymectomized mice would reflect a selective increase in one of the two major regulatory subsets (thymic‐ vs. peripherally differentiated Treg cells). Thymus removal might impact peripheral Treg cell composition in different ways, since exportation of both newly differentiated tTreg and conventional CD4^+^ RTE (the major source of pTreg in vivo) are blocked. However, the percentage of neuropilin‐1^+^ among Foxp3^+^ cells was not modified in thymectomized animals, suggesting that thymic dysfunction does not significantly impact the peripheral tTreg/pTreg ratio (Fig. 1H). Thus, thymectomy of young animals reproduces the disturbed peripheral T cell homeostasis of aged mice.

### Grafting of aged mice with young thymi restores the Treg/Tconv balance

**Figure 2:**
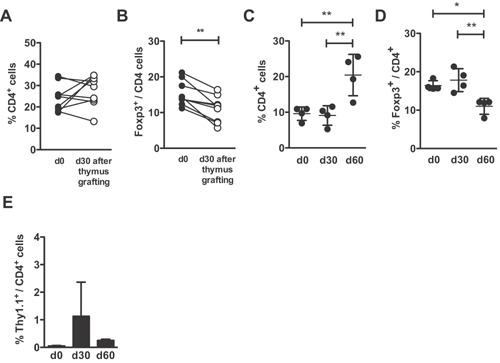
Grafting of a functional thymus onto aged mice restores their physiological peripheral frequencies of CD4^+^ and Treg cells BALB/c or C57BL/6 mice were grafted, at the age of 18 months, with one syngeneic thymus harvested from 5 to 10 days old donor, and alterations in their peripheral frequencies of CD4^+^ and CD4^+^Foxp3^+^ were determined by flow cytometry. (A, C) CD4^+^ and (B, D) Foxp3^+^/CD4^+^ T cell frequencies are shown in the peripheral blood before, 30 and 60 days after thymus grafting of (A, B) BALB/c hosts and (C, D) C57BL/6 hosts. (E) CD4^+^Thy1.1^+^ B6Ba donor cell frequencies in the peripheral blood before, 30 and 60 days after thymus grafting in C57BL/6 hosts. Each circle represents one individual mouse. Data are pooled from three independent experiments and statistical significance was determined by paired Student's *t*‐test (A and B) and one‐way ANOVA with Bonferroni's Multiple Comparison Test (C–E) (*p < 0.05, **p < 0.01, ***p < 0.001; ns, not significant).

To confirm the above observation, we performed the grafting of a functional young thymus into aged hosts. Thymuses from 5 to 10 days old donors were used indiscriminately, since they are easier to graft in the renal capsule than older thymuses and, as already shown 33, in the 5–10 days of age interval they do not present important qualitative differences in Tregs. Also, at the time we analyzed the effects on the host T cell populations, these grafted thymuses, aged at least 30–35 days, are qualitatively similar in their T cells export. This manipulation was able to restore the peripheral CD4^+^ T cell levels within the first two months after grafting and the CD4^+^Foxp3^+^ Treg cell frequency was significantly diminished in all grafted animals in both BALB/c and B6 strains (Fig. 2A–D). Note that aged B6 mice display, two months after being grafted with functional thymuses, CD4 and Treg cell levels comparable to those detected in young animals (see Fig. S1B for comparison). These data support the hypothesis that thymus export is the major regulator of peripheral T cell homeostasis, regardless of animal age. Interestingly, the recovery of the CD4^+^ T cell frequencies does not appear to be a consequence of the prevalent incorporation of a single cohort of young RTE cells already present in the grafted thymus, as no more than 1% of donor‐derived Thy1.1^+^ CD4^+^ cells were seen at 1 and 2 months after thymus grafting (Fig. 2E).

In addition to the observed rise in peripheral CD25^+^Foxp3^+^ Treg cells, aged mice had also an increased frequency of activated CD25^+^Foxp3^−^ effector T (Teff) lymphocytes. Consequently, when compared to young mice, they showed a significantly lower ratio of Treg/Teff (Fig. S3). The higher level of Treg cells in aged animals may be, thus, insufficient to control the pool of CD25^+^ activated T cells. Grafting of aged mice with a young thymus resulted in an even lower Treg/Teff ratio, since the correction of the Treg frequency was not accompanied by a diminished level of activated lymphocytes. Thymectomized mice, on the contrary, did not present an enhanced level of activated conventional T cells. Overall, these results suggest that the dynamics of the activated T cell pool is regulated independently of thymic output.

### Aged progenitors are able to efficiently colonize and differentiate in a young thymus graft

**Figure 3:**
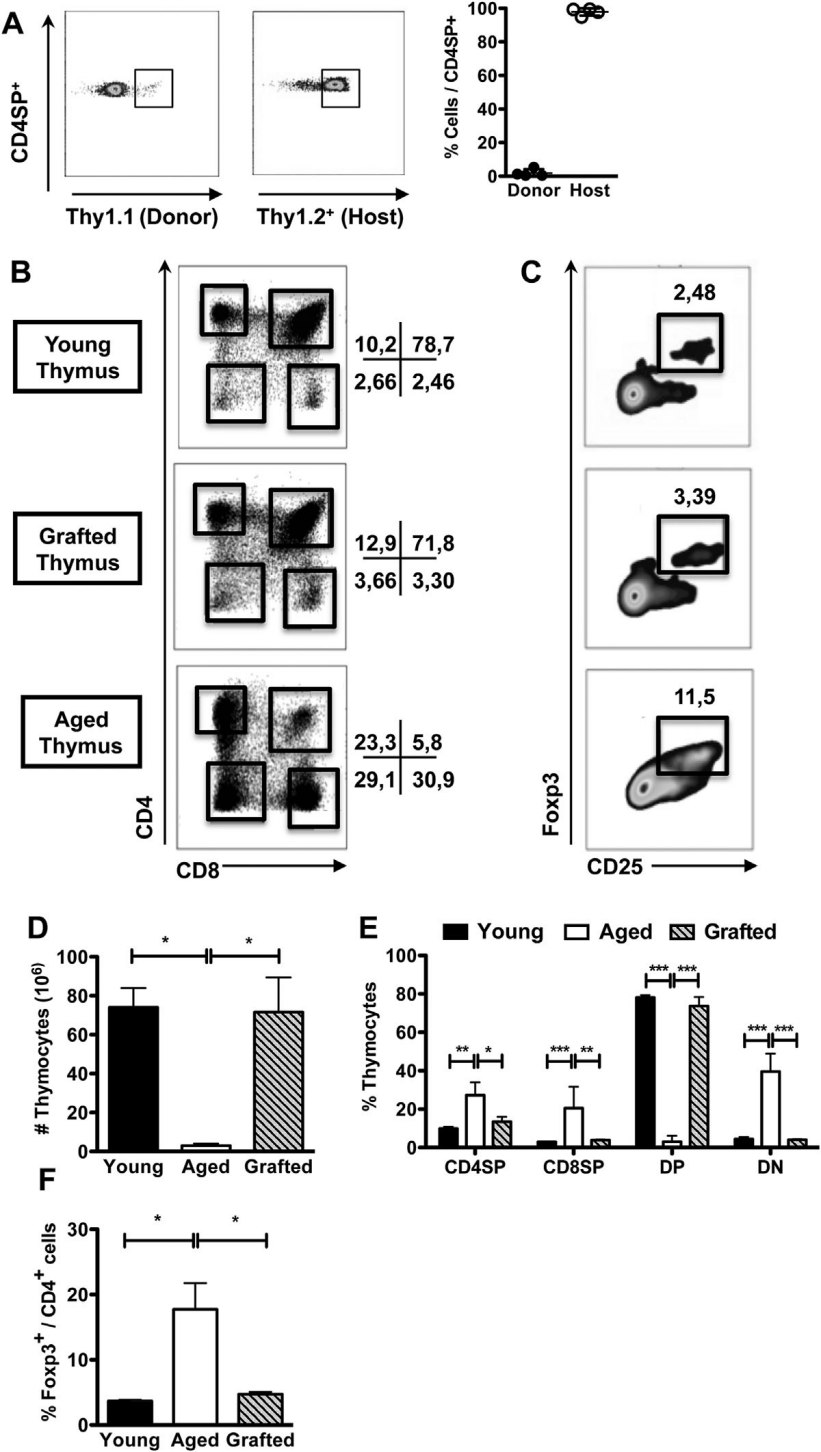
Aged precursors are able to differentiate in the grafted young thymus and generate thymic subsets at normal frequencies. BALB/c or C57BL/6 mice were grafted, at the age of 18 months, with one syngeneic thymus obtained from 5 to 10 days old donor. (A) Representative dot‐plots (left) show the Thy1.1 and Thy1.2 staining gated on single‐positive CD4^+^ (CD4SP) T lymphocytes. Scatter‐plot graph (right) shows the relative frequencies of B6Ba Thy1.1^+^ donor‐ and B6 Thy1.2^+^ host‐derived thymocytes among CD4SP present in the grafted thymuses (*n* = 4) 30 days after grafting. (B, C) Representative dot‐plots depict frequencies of (B) CD4^+^ and CD8^+^ and (C) CD25^+^ and Foxp3^+^ (gated on CD4SP) thymocytes present in the grafted thymuses of BALB/c mice. The same staining is shown in control BALB/c young (2 months old) and in the endogenous aged BALB/c host thymuses for comparison. (D) Absolute number of total thymocytes, (E) percentages of CD4SP, CD8^+^ single‐positive (CD8SP), CD4^+^CD8^+^ double‐positive (DP), CD4^−^CD8^−^ double‐negative (DN), and (F) Foxp3^+^/CD4^+^ cells (among CD4SP lymphocytes) are shown in the grafted thymuses (*n* = 3) and compared with control young (*n* = 3) and endogenous aged host (*n* = 3) thymuses of BALB/c mice. Each circle represents one individual mouse. Data are representative from three independent experiments with similar results and statistical significance was determined by one‐way ANOVA with Bonferroni's Multiple Comparison Test (D and F) and two‐way ANOVA with Bonferroni's Multiple Comparison Test (E) (*p < 0.05, **p < 0.01, ***p < 0.001, ns, not significant).

In order to investigate whether the young thymic graft would be able to support normal T cell differentiation from aged hematopoietic precursors, thymi from young B6Ba Thy1.1^+^ mice were grafted into B6 Thy1.2^+^ aged hosts. One month after grafting, almost all T cells present in the Thy1.1^+^ donor‐derived grafted thymus were Thy1.2^+^ (Fig. 3A), showing that aged host‐derived precursors were able to colonize and differentiate into mature lymphocytes in a younger thymus. Among them, the CD4^+^, CD8^+^, and CD25^+^Foxp3^+^ T cell subpopulations were quantitatively very similar to those found in a thymus isolated from a non‐manipulated young mouse (Fig. 3B–F). Thymocyte cellularity (Fig. 3D) and the frequencies of DN (double‐negative), DP, CD4SP (CD4 single‐positive), and CD8SP (CD8 single‐positive) (Fig. 3E,F) were maintained unaltered in the young grafted thymus after its insertion into the aged host environment and colonization by aged precursors. The number of CD25^+^Foxp3^+^ thymocytes was also essentially the same as that present in a control young thymus (Fig. 3C,F). In contrast, the aged host thymus maintained its typically altered pattern for each thymocyte population (Fig. 3B–F), without any dominant influence of the young thymus graft. A very low frequency of DP (indicative of poor T cell maturation), in parallel with higher frequencies of DN, CD4SP, and CD8SP cells, were present in the host atrophied thymus, together with an increased Foxp3^+^ cell frequency among CD4SP thymocytes. The maintenance of an intact defective host aged thymus may weaken the need for a control using aged thymus grafts, a technically very hard procedure. Our results strongly indicate that the preservation of thymic function, through the production of normal levels of mature T lymphocytes, is sufficient to overcome the age‐related alterations in T cell homeostasis, regardless of the senescence of the peripheral compartment.

### The CD25^neg/low^Foxp3^+^ Treg cells are preferentially altered in aged and thymectomized mice

**Figure 4:**
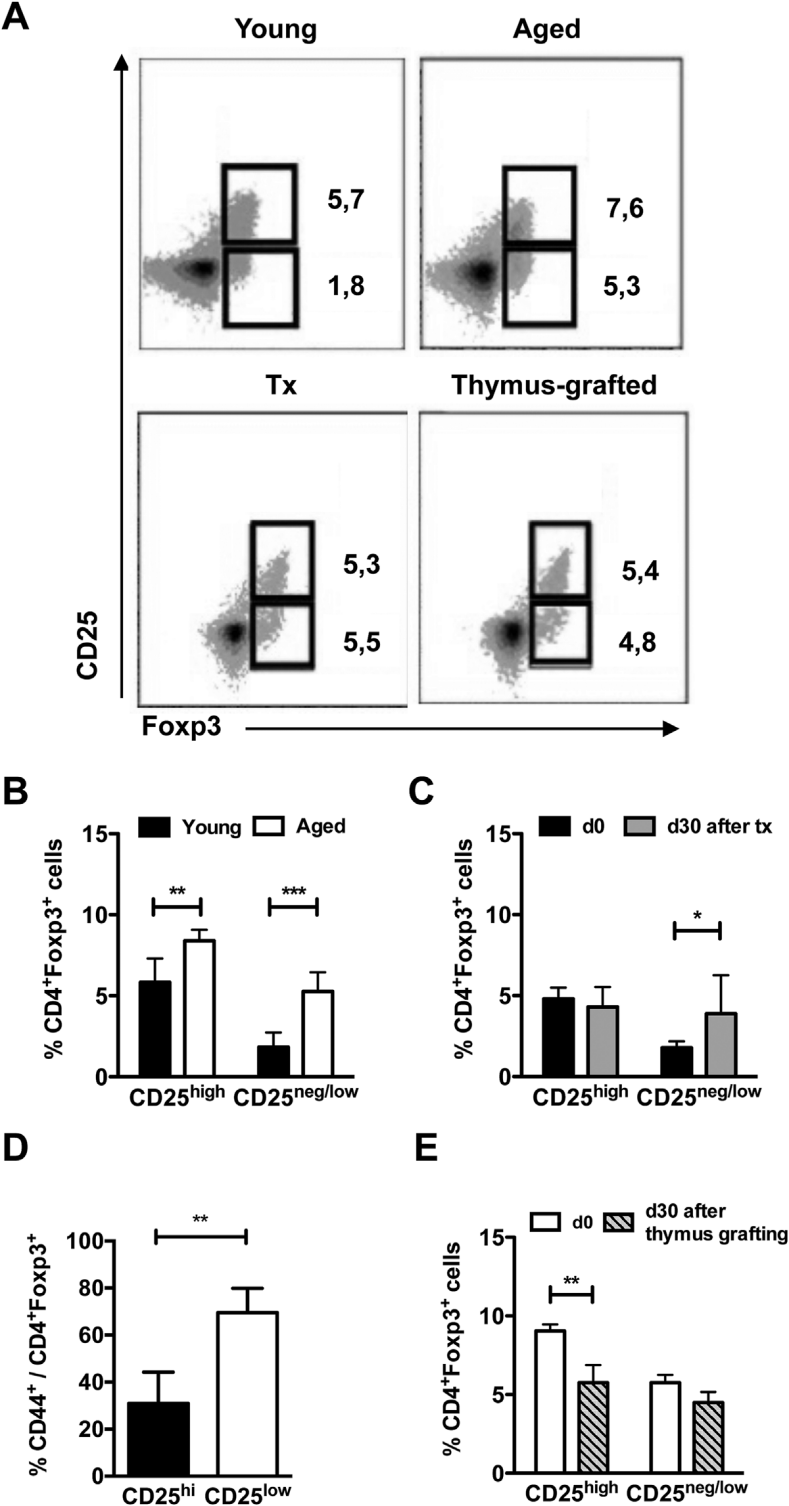
Frequencies of CD25^low^Foxp3^+^cells are preferentially altered in thymectomized mice. The frequencies of Foxp3^+^CD25^high^ and Foxp3^+^CD25^low^ cells in the blood of young (2 months old, *n* = 8), aged (18 months old, *n* = 8), young mice thymectomized 30 days before (Tx, *n* = 6) and aged BALB/c mice grafted with a young syngeneic thymus 30 days before (*n* = 6) were determined by flow‐cytometry. (A) Representative dot‐plots are shown. (B, C, E) Column bar graphs show the percentages of CD25^high^ and CD25^neg/low^ events among Foxp3^+^ cells from (B) young and aged mice, (C) young mice before and 30 days after thymectomy, and (E) aged mice before and 30 days after young thymus grafting. (D) Frequencies of CD44^+^ cells among CD25^high^ and CD25^neg/low^ Treg cells 30 days after thymectomy. Data are pooled from three independent experiments and statistical significance was determined two‐way ANOVA with Bonferroni's Multiple Comparison Test (B and C), Unpaired t test (D), and two‐way ANOVA with Bonferroni's Multiple Comparison Test by Student's *t*‐test (E) (*p < 0.05, **p < 0.01, ***p < 0.001, ns, not significant).

The peripheral CD4^+^Foxp3^+^ Treg cell population from young individuals comprises a minor fraction (about 2%) of CD25^neg/low^ cells, proposed to be prone to lose Foxp3 expression under inflammatory conditions 34. The augment in the CD4^+^Foxp3^+^ T cell frequency of aged animals is more prominent in this fraction (Fig. 4A,B). Similarly, in the thymectomized young animals, only the CD25^neg/low^Foxp3^+^ cell frequency was significantly enlarged (Fig. 4A,C), most of them showing a CD44^high^ phenotype (Fig. 4D). Grafting of a young thymus into aged hosts, on the other hand, preferentially affected the CD25^high^Foxp3^+^ cells, restoring their numbers to values approaching those found in young mice (Fig. 4A,E).

### Treg and Tconv of different ages are equally able to colonize the peripheral compartment

**Figure 5:**
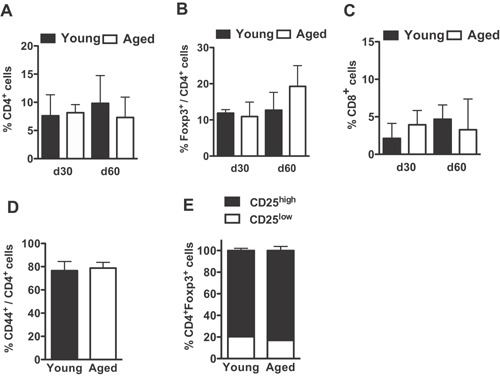
Aged and young T lymphocytes present similar ability to colonize the periphery when transferred to lymphopenic hosts. Young B6.RAG2^−/−^ mice were adoptively transferred with unfractionated splenocytes from young (2 months old) or aged (18 months old) B6.Ba mice and the frequencies of T lymphocyte subpopulations were determined, by flow‐cytometry, in the peripheral blood 1 and 2 months after reconstitution. (A–C) Column bar graphs compare the percentages of (A) CD4^+^, (B) CD4^+^Foxp3^+^, and (C) CD8^+^ T cells found in hosts receiving young (*n* = 5) or aged (*n* = 5) donor splenocytes. (D–E) Frequencies of (D) CD44^+^ among CD4^+^ cells and (E) CD25^low^ and CD25^hi^ among CD4^+^Foxp3^+^ cells at 1 month after transfer (*n* = 5). Data are pooled from two independent experiments and statistical significance was determined by one‐way ANOVA with Bonferroni's Multiple Comparison Test (A–C) and unpaired student's *t*‐test (D) (*p < 0.05, **p < 0.01, ***p < 0.001).

The progressive rise in Treg cell frequencies after natural or experimental reduction/interruption of thymus output might derive from an enhanced competence of Treg cells to persist in the peripheral compartment. In order to verify if the survival and colonization potential of Treg and Tconv lymphocytes varies with age, we independently transferred young and aged splenocytes, normalized to contain the same absolute number of CD4^+^Foxp3^+^ cells in the inocula, to lymphopenic RAG^−/−^ hosts. Similar frequencies of Treg/Tconv lymphocyte subpopulations were found in the hosts receiving young or aged splenocytes at one and two months after transfer (Fig. 5A–D). Equivalent proportions of CD25^high^/CD25^neg/low^ among Foxp3^+^ cells were also found in both groups (Fig. 5E).

An increased percentage of total Treg cells, in comparison to the physiologic Treg cell frequency observed in euthymic mice was found in all animals, regardless the age of the donor population (Fig. 5). Therefore, the absence of thymus export, not the age of the peripheral environment or the age of T lymphocytes, is the crucial factor determining the peripheral accrual of Treg cells.

## Discussion

The peripheral T cell compartment of aged individuals is characterized by great modifications, including a higher frequency of Treg cells 21, 22, 25, 26, 30, 31, 32. The relative contribution of alterations in thymic exportation versus changes in the homeostasis of the peripheral compartment affecting the Treg/Tconv lymphocytes balance is not yet clearly established. Controversial results regarding changes in the proportion of different thymocyte subpopulations, as well as on the efficiency of peripheral conversion to the Treg phenotype in aged versus young individuals, were already reported 23, 27, 31.

Concerning the alterations in thymus subsets, we and others 27 observed a higher percentage of Foxp3^+^ lymphocytes in the aged thymus. It has not been verified, however, if this enhanced Treg cell frequency is maintained in the peripheral RTE population, indicative of their higher output in comparison to the younger thymus. The relative contribution of differentiation in situ and/or accumulation of recirculating cells for the reported augmented number of Treg cells present in the aged thymus is also obscure. As recently shown 35, long‐lived Treg cells accumulate in the thymus with age, as a result of recirculation of mature Treg cells into the thymus, and of their subsequent retention in this organ. These Treg cells retained in the thymus were shown to be responsible for an inhibitory effect in the generation and exportation of new Treg cells 36. Therefore, it seems unlikely that most Treg cells found in the aged thymus are newly differentiated, in agreement with the dysfunctional behavior of the involuted thymus.

Actually, Chougnet et al. 22 verified that the Treg/Tconv balance in the small population of T lymphocytes emerging from the thymus is not altered in aged mice. These authors also reported a lower expression of Bim and higher resistance to apoptosis in aged peripheral Treg cells, which they considered responsible for their intense accrual. More recently, this same group also showed a role for IL‐6 and ICOS expression in promoting Treg cell frequency alteration with age 26. Changes in other populations from the aged thymus, however, might also influence the peripheral homeostasis of different T cell subsets and indirectly modify Treg cell frequency. Several alterations are seen in parallel to thymus involution, such as degeneration of T cell progenitors, deterioration of the thymic stroma, and alterations in miRNA expression in thymic epithelial cells (TEC) 37, 38.

We showed here that the interruption of thymic output in mice thymectomized at the age of 4–6 weeks resulted in a progressive increase of peripheral Treg cell frequencies, in parallel to decreased total CD4^+^ cell proportions, simulating the alterations described for natural thymic involution occurring in aged mice. The enhancement in Treg cell frequency was very quick, being detected as early as one month after surgery. Therefore, the Treg cells present in the periphery of a young mouse are able to persist in a higher proportion than the conventional T cells after thymectomy, leading to their favored accumulation. This result strongly suggests that the rise in the Treg cell frequency is not dependent on intrinsic properties of aged peripheral Treg cells, such as lower expression of Bim or reduced susceptibility to apoptosis, which were described as significantly modified only in mice older than 10 months old 22.

This rapid augment in the percentage of peripheral Treg cells when thymus is removed is in accordance with observations made by our group 28, 29 on an enhanced accrual of Treg cells when total lymphoid populations are transferred to lymphopenic hosts. A higher percentage of total Treg cells, around 20% of peripheral CD4^+^ T cells, is normally obtained after transfer of young‐derived splenic or thymus cell suspensions to athymic nude hosts or RAG^−/−^ mice. Here, we confirmed that the colonization of the peripheral compartment by aged‐derived splenic CD4^+^ T lymphocytes gives rise to Treg cell frequencies indistinguishable from those previously reported for young‐derived cells. These findings suggest that, in the absence of thymic output, Treg cells, independently of donor age, are fitter to expand and/or to survive in the peripheral compartment in comparison to Tconv cells. Accordingly, a lower percentage of annexin‐V‐positive cells were observed in the Treg cell subset, in comparison to the Tconv lymphocytes, when transferred to lymphopenic hosts 28 or in thymectomized mice. Notably, this altered peripheral homeostasis in the absence of thymus output is also not influenced by the age of the peripheral compartment of the host. Consistent to our data, Bourgeois et al. 39, using a model of chemical thymectomy by inducible RAG ablation, have also described a selective decay in the naïve peripheral T cell compartment, and not in the regulatory or the memory T cell subsets after interruption of thymus output.

Different results were recently reported using a protocol of accelerated thymic involution in a Foxn1 conditional knockout mouse 40. Although an increased frequency of Treg lymphocytes was observed inside the atrophied thymus of the Foxn1‐cKO mice, there were no alterations in the peripheral frequencies of this population, so the authors concluded that the young environment could reverse age‐related Treg cell accumulation. However, in this work, thymic and peripheral Treg cell frequencies were only evaluated at 2–3 weeks after the induction of thymic involution. Peripheral alterations might be detectable at a longer time interval, especially if they depend on a prolonged survival of Treg cells. Another possible explanation for the discrepancy in the results may be related to the use of tamoxifen, an estrogen‐related drug, to induce thymus atrophy at a defined life period. Unanticipated hormonal effects consequent to this treatment may have influenced peripheral T cell homeostasis in an unknown proportion. In accordance with our results, these authors found a similar increase in Treg cell frequency when splenocytes were transferred to RAG^−/−^ mice, independently of the donor age, confirming that Treg cells are more efficient in peripheral colonization and persistence than Tconv lymphocytes.

The higher prevalence of Treg cells obtained after thymectomy or after transfer to a lymphopenic host could also derive from an enhanced conversion of Tconv lymphocytes to the Treg phenotype. A lower rate of this conversion might be expected for aged populations, since RTE lymphocytes, the most efficient precursors for pTregs 24, constitute a minor population in aged animals. On the other hand, the few naïve T cells leaving the aged thymus encounter a peripheral compartment extremely depleted of naïve T lymphocytes, simulating a lymphopenic condition where an excess of homeostatic cytokines might enhance the rate of pTreg cell induction. As already reported 18, aged CD4^+^ Tconv cells present an intrinsic inability to convert to pTreg cells. This failure was not seen in the same work, however, for CD4^+^ Tconv cells from young mice previously thymectomized, suggesting that the defective Treg induction from aged CD4^+^ cells in the periphery is an exclusive property of the aged mature CD4^+^ precursors. In lymphopenic hosts transferred with thymocytes or splenocytes, we observed low frequencies of Nrp‐1^−^Foxp3^+^ T cells, similar to those present in control euthymic mice 28. In thymectomized mice, we also observed a low frequency of these Nrp‐1^−^ Treg cells, without significant changes in comparison to lymphoreplete animals. The increased frequency of Foxp3^+^ cells when lymphoid populations expand in the absence of the thymus apparently does not involve, thus, a more intense peripheral conversion to the Treg phenotype.

A return to the physiological frequency of Treg cells, similar to that present in young mice, was also very promptly obtained in old animals after grafting with a young thymus. This restoration of normal Treg/Tconv lymphocyte proportions, verified as soon as 1 month after grafting, comprised only a peripheral adjustment, since no changes were seen in the original proportions of thymic subpopulations belonging to the young grafted thymus or to the aged host thymus. The aged peripheral microenvironment is, thus, not capable of interfering in the frequencies of DP, CD4SP, CD8SP and Foxp3^+^ thymocytes described for a young thymus and, more importantly, is not sufficient per se to promote aged‐related shifts in the T cell homeostasis. It is also interesting to observe that the aged T cell precursors are fully able to colonize and differentiate in the young grafted thymus, which was almost entirely comprised of Thy1.2^+^ host‐derived cells after 1 month. These results suggest that the continuous output of the young grafted thymus, which is numerically much superior to the small number of cells emigrating from the aged host thymus, may contribute to normalize the peripheral proportions of Treg/Tconv cells. The aged peripheral compartment does not interfere with this homeostasis.

Among the changes in the Treg cell population of aged and thymectomized animals, we found a preferential increase in the CD25^neg/low^‐expressing Foxp3^+^ subset, consistent to what was already reported for aged animals 21, 25. This population was described as being involved in the pathogenesis of different autoimmune diseases, due to harboring an elevated frequency of autoreactive lymphocytes and being more prone to lose Foxp3 expression under inflammatory settings 3. We observed that the majority of these cells also express CD44 before and after the thymectomy, suggesting enrichment in activated lymphocytes. If these cells are particularly fitter to persist in the periphery, they might lead to the increased occurrence of autoimmunity in aged individuals.

The grafting of a young thymus in the aged host led to a preferential decrease in the frequency of CD25^high^Foxp3^+^ cells, suggesting that they are less resistant to changes in peripheral homeostasis. It is possible that the preservation of the CD25^neg/low^Foxp3^+^ T cells is derived from their continuous stimulation in the periphery and their lower Bim expression, as described by Raynor et al. 25.

Besides the increase in Treg cell frequency, the percentage of activated T cells is also augmented over aging, resulting in a diminished ratio of regulatory/activated lymphocytes in the periphery. This excess of activated effector T cells may be responsible for the higher frequency of autoimmune diseases, in spite of the enrichment in peripheral Treg cells, and may contribute to the elevated concentration of inflammatory cytokines described for the elderly 41, 42, 43. On the other hand, the decreased ratio of naïve Tconv/Treg lymphocytes may lead to the impaired response of older individuals to new stimulus associated with infections, tumors, or vaccines, characterizing an immunodeficient state 30, 44.

Our results, thus, highlight the importance of the thymus as a permanent source of emigrating populations of recently differentiated lymphocytes harboring an adequate, physiological proportion of Treg/Tconv lymphocytes, essential to keep the peripheral Treg cell balance, regardless of the aging status of the peripheral compartment.

## Conflict of Interest

None declared.

## Supporting information

Additional supporting information may be found in the online version of this article at the publisher's web‐site.

**Figure S1**. Peripheral frequencies of CD4+, Foxp3+/CD4+ and activated T cells are altered in aged mice.
**Figure S2**. Absolute numbers of CD4+ T cells are decreased, while CD4+Foxp3+ Treg cells are unchanged, in the blood of thymectomized mice.
**Figure S3**. The frequency of CD4+CD25+Foxp3‐ effector T cells is also increased in aged mice and the Treg/Teff cell ratio is not altered.

Supporting Figure Legends
