# Supplementary figures and images for: Critical influence of the thymus on peripheral T cell homeostasis
Source: Immun Inflamm Dis. 2016 Nov 28;4(4):474–86. doi: 10.1002/iid3.132 (PMC5134722; doi:10.1002/iid3.132)

Supporting Figure 1

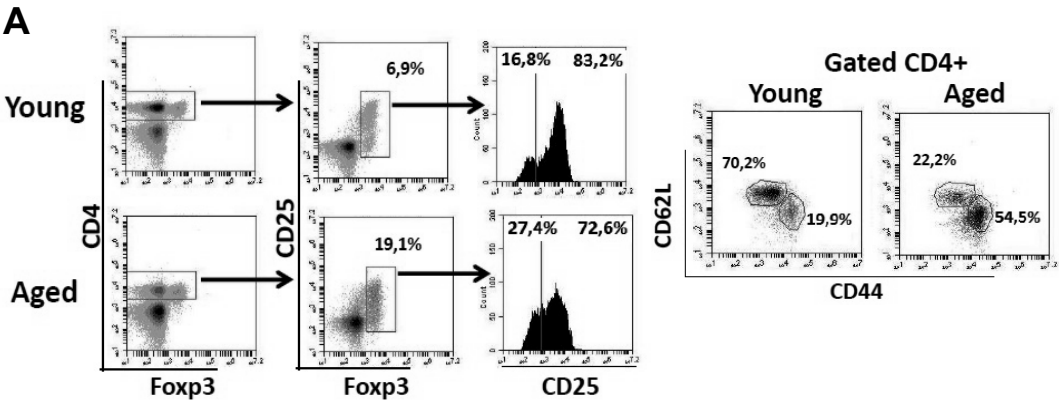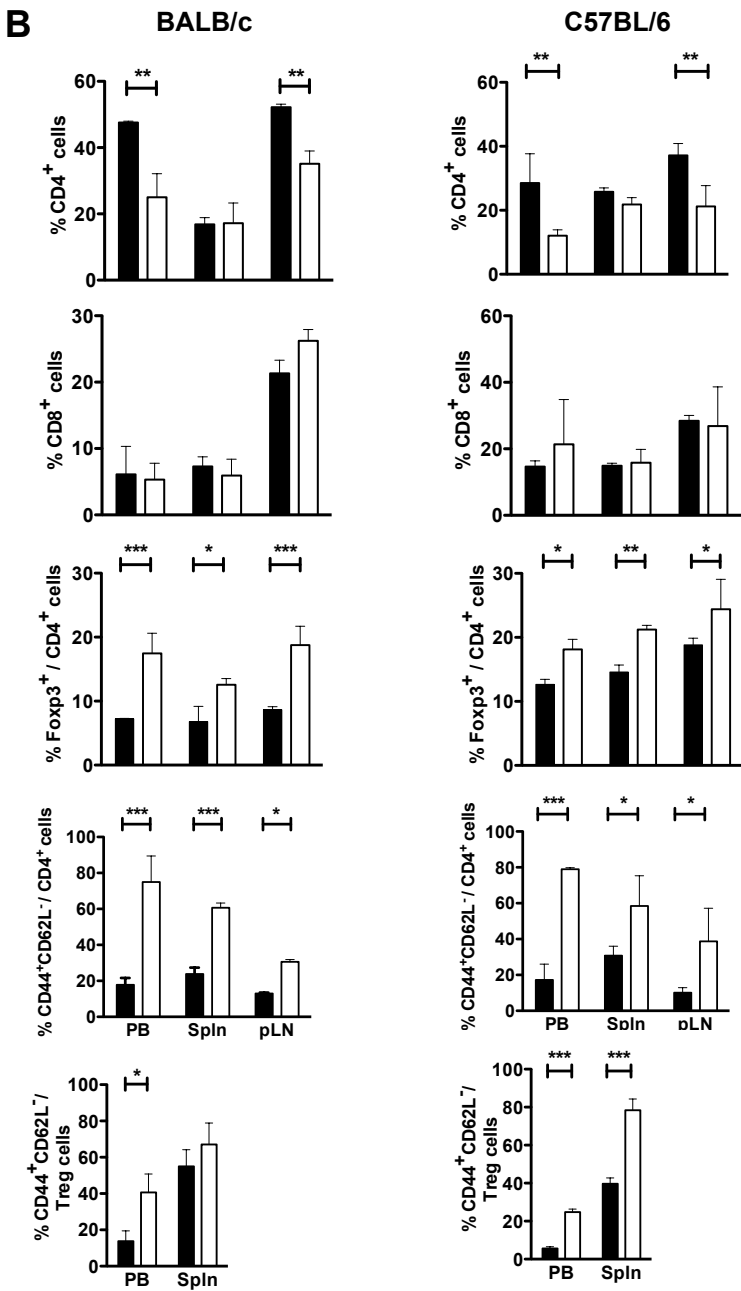

Supporting Figure 2

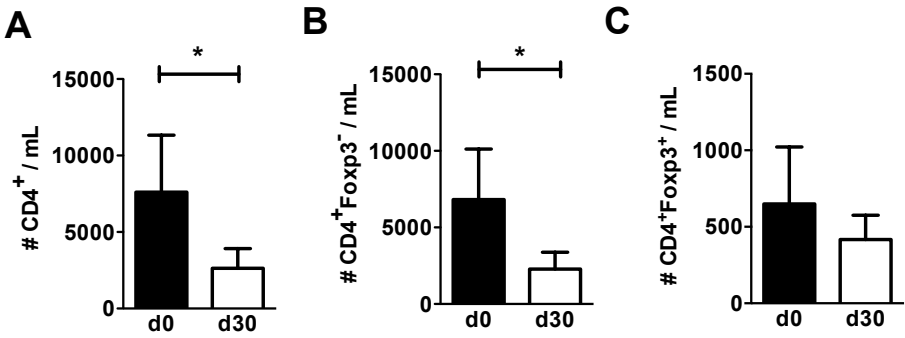

Supporting Figure 3

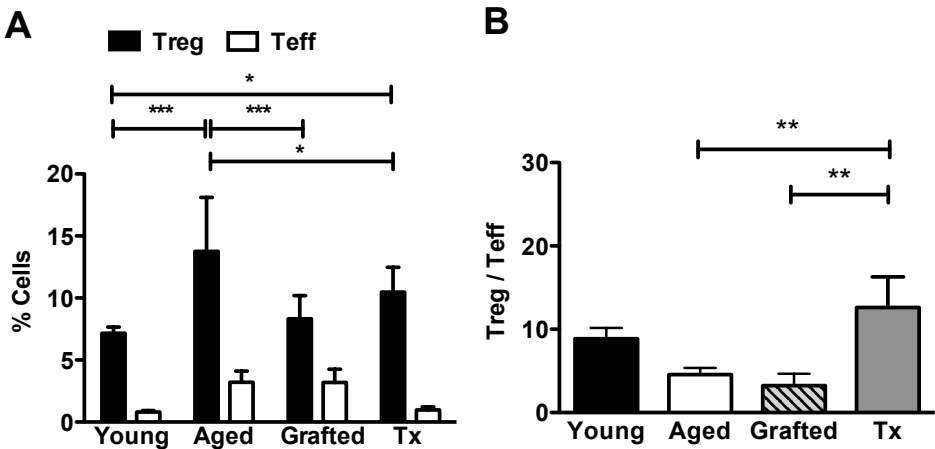

Supplement: Supplementary file 1 — Figure S1. Peripheral frequencies of CD4+, Foxp3+/CD4+ and activated T cells are altered in aged mice. Figure S2. Absolute numbers of CD4+ T cells are decreased, while CD4+Foxp3+ Treg cells are unchanged, in the blood of thymectomized mice. Figure S3. The frequency of CD4+CD25+Foxp3‐ effector T cells is also increased in aged mice and the Treg/Teff cell ratio is not altered. [file IID3-4-474-s001.pdf]
