# Supplementary material for: Critical influence of the thymus on peripheral T cell homeostasis
Source: Immun Inflamm Dis. 2016 Nov 28;4(4):474–86. doi: 10.1002/iid3.132 (PMC5134722; doi:10.1002/iid3.132)
Supplement: Supplementary file 2 — Supporting Figure Legends [file IID3-4-474-s002.pdf]

Critical influence of the thymus on peripheral T cell homeostasis

Pedro Henrique Oliveira Vianna[1,2], Fábio Barrozo do Canto[1,2], Jeane de Souza Nogueira[1,2], Caroline Fraga Cabral Gomes Nunes[1,2], Adriana César Bonomo[3], Rita Fucs[2]

[1]Departamento de Imunologia, Instituto de Microbiologia Paulo de Goés (IMPG) – Universidade Federal do Rio de Janeiro, Rio de Janeiro - RJ, Brazil. [2]Departamento de Imunobiologia, Instituto de Biologia – Universidade Federal Fluminense, Niterói - RJ, Brazil. [3]Programa FIOCANCER, VPPLR – Instituto Oswaldo Cruz - FIOCRUZ, Rio de Janeiro - RJ, Brazil

Supporting Information

Figure S1: **Peripheral frequencies of $CD4^+$, $Foxp3^+/CD4^+$ and activated T cells are altered in aged mice.** Frequencies of $CD4^+$, $CD8^+$, $Foxp3^+/CD4^+$ and $CD62L^-CD44^+/CD4^+$ T cells were determined, by flow cytometry, in the blood, spleen and peripheral lymph nodes of young (2-3 months-old) and aged (>18 months-old) mice. (A) Representative dot-plots show the gating strategy. Scatter-plot graphs depict the percentages of peripheral populations in (B) BALB/c mice (n=6) and (C) C57BL/6 mice (n=6). Data are from three independent experiments and statistical significance was determined by Two-way ANOVA with Bonferroni`s Multiple Comparison Test (B) (*p<0.05, **p<0,01***p<0,001).

Figure S2: Absolute numbers of $CD4^+$ T cells are decreased, while $CD4^+Foxp3^+$ Treg cells are unchanged, in the blood of thymectomized mice. Absolute numbers of $CD4^+$, $CD4^+Foxp3^+$ and $CD4^+Foxp3^-$ cells were determined by flow-cytometry. Column bar graphs show the number of (A) $CD4^+$, (B) $CD4^+Foxp3^-$ and (C) $CD4^+Foxp3^+$ cells in blood samples of young mice 30 days after thymectomy (Tx, n=4). Data are from one experiment and statistical significance was determined by unpaired Student's t test (B and C) (*p<0.05, **p<0,01***p<0,001).

Figure S3: **The frequency of $CD4^+CD25^+Foxp3^-$ effector T cells is also increased in aged mice and the Treg/Teff cell ratio is not altered.** (A) Column bars show the the percentage of Treg ($CD4^+CD25^+Foxp3^+$) and Teff ($CD4^+CD25^+Foxp3^-$) determined by flow-cytometry in the blood of young mice (2 months-old, n=4), aged mice (18 months old, n=4), aged mice 30 days after grafting with an young thymus (Grafted, n=4) and young mice 30 days after thymectomy (Tx, n=4). (B) Column bars show the ratio Treg/Teff among $CD4^+$ Tcells in each group. Data are pooled from two independent experiments and statistical significance was determined by Two-way ANOVA with Bonferroni`s Multiple Comparison Test (A) and One-way ANOVA with Bonferroni`s Multiple Comparison Test t (B) (*p<0.05, **p<0,01***p<0,001).
